# Evaluation der Einführung der HIV-Präexpositionsprophylaxe als Leistung der gesetzlichen Krankenversicherung (EvE‑PrEP): Hocheffektiver Schutz vor HIV und keine Zunahme von sexuell übertragbaren Infektionen

**DOI:** 10.1007/s00103-023-03733-0

**Published:** 2023-07-12

**Authors:** Daniel Schmidt, Christian Kollan, Knud Schewe, Nikola Hanhoff, Robin Rüsenberg, Martin Friebe, Tim Schikowski, Susanne Barbara Schink, Ulrich Marcus, Uwe Koppe, Klaus Jansen, Hendrik Streeck, Patrick Ottensmeyer, Matthias an der Heiden, Norbert Bannert, Rosaline M’Bayo, Maia Ceres, Lukas Weber, Holger Sweers, Axel Jeremias Schmidt, Helge Tietz, Emmanuel Danan, Jörg Bendig, Stephan Kloep, Anja Neumann, Frederik Valbert, Jürgen Wasem, Barbara Bartmeyer, Viviane Bremer, NEPOS Studiengruppe

**Affiliations:** 1 Fachgebiet HIV/AIDS und andere sexuell oder durch Blut übertragbare Infektionen, Abteilung für Infektionsepidemiologie, Robert Koch-Institut Berlin, Berlin, Deutschland; 2 Deutsche Arbeitsgemeinschaft ambulant tätiger Ärztinnen und Ärzte für Infektionskrankheiten und HIV-Medizin e. V. (dagnä), Berlin, Deutschland; 3 Fachgebiet Nosokomiale Infektionen, Surveillance von Antibiotikaresistenz und -verbrauch, Robert Koch-Institut Berlin, Berlin, Deutschland; 4 Institut für Virologie, Universitätsklinikum Bonn, Bonn, Deutschland; 5 Abteilung für Infektionskrankheiten, Robert Koch-Institut Berlin, Berlin, Deutschland; 6 Afrikaherz/Verband für Interkulturelle Arbeit Berlin/Brandenburg e.V. (VIA), Berlin, Deutschland; 7 Berufsverband erotische und sexuelle Dienstleistungen e. V. (BesD), Köln, Deutschland; 8 HILFE-FÜR-JUNGS e. V./subway, Berlin, Deutschland; 9 Abteilung Öffentlichkeitsarbeit und Crossmediale Kommunikation, Deutsche Aidshilfe e. V. (DAH), Berlin, Deutschland; 10 Fachbereich Medizin und Gesundheitspolitik, Deutsche Aidshilfe e. V. (DAH), Berlin, Deutschland; 11 Checkpoint BLN/PrEP-User, Berlin, Deutschland; 12 prep.jetzt, Berlin, Deutschland; 13 Kompetenzzentrum für Klinische Studien, Universität Bremen (KKSB), Bremen, Deutschland; 14 Lehrstuhl für Medizinmanagement, Universität Duisburg-Essen, Essen, Deutschland

**Keywords:** HIV-Prävention, Evaluation, Zugangsbarrieren, Gesetzliche Krankenversicherung (GKV), Deutschland, HIV prevention, Evaluation, Access barriers, Statutory health insurance (SHI), Germany

## Abstract

**Hintergrund:**

Untersucht wurden die Auswirkungen der HIV-Präexpositionsprophylaxe (PrEP) als neue Leistung der gesetzlichen Krankenversicherung (GKV) auf das Infektionsgeschehen von HIV und anderen sexuell übertragbaren Infektionen (STI) in Deutschland. Zusätzlich wurden PrEP-Bedarfe sowie Zugangsbarrieren analysiert.

**Methoden:**

Im Rahmen des Evaluationsprojektes wurden folgende Daten ausgewertet: HIV- und Syphilis-Meldedaten und erweiterte Surveillance des Robert Koch-Instituts (RKI), Apothekenabrechnungsdaten, GKV-Daten, PrEP-Gebrauch in HIV-Schwerpunktzentren, Checkpoint‑, BRAHMS- und PrApp-Studie sowie ein Community-Beirat.

**Ergebnisse:**

Die PrEP-Nutzenden waren zum größten Teil männlich (98–99 %), zum Großteil im Alter zwischen 25–45 Jahren und überwiegend deutscher Staatsangehörigkeit oder Herkunft (67–82 %). Der Großteil zählte zur Gruppe der Männer, die Sex mit Männern haben (99 %). In Bezug auf HIV-Infektionen zeigte sich die PrEP als hocheffektiv. Es fanden nur vereinzelt HIV-Infektionen statt (HIV-Inzidenzrate 0,08/100 Personenjahre), in den meisten Fällen war der vermutete Grund eine geringe Adhärenz. Die Inzidenzen von Chlamydien, Gonorrhö und Syphilis nahmen nicht zu, sondern blieben nahezu gleich oder gingen sogar zurück. Es zeigte sich Informationsbedarf zur PrEP für Personen in trans*/nichtbinären Communitys, Sexarbeitende, Migrant*innen und Drogengebrauchende. Notwendig wären bedarfsgerechte Angebote für Zielgruppen mit erhöhtem HIV-Risiko.

**Diskussion:**

Die PrEP erwies sich als eine sehr wirkungsvolle HIV-Präventionsmethode. Die teils befürchteten indirekten negativen Einflüsse auf STI-Raten wurden in dieser Untersuchung nicht bestätigt. Aufgrund der zeitlichen Überschneidung mit den Eindämmungsmaßnahmen während der COVID-19-Pandemie wäre für eine abschließende Beurteilung ein längerer Beobachtungszeitraum wünschenswert.

## Abstract (en)

**Background:**

We investigated the impact of HIV pre-exposure prophylaxis (PrEP) as a new service of the statutory health insurance (SHI) on the incidence of HIV and other sexually transmitted infections (STIs) in Germany. In addition, PrEP needs and access barriers were analyzed.

**Methods:**

The following data were evaluated as part of the evaluation project: HIV and syphilis notification data and extended surveillance by the Robert Koch Institute (RKI), pharmacy prescription data, SHI routine data, PrEP use in HIV-specialty care centers, Checkpoint, the BRAHMS and PrApp studies, as well as a community board.

**Results:**

The majority of PrEP users were male (98–99%), primarily aged between 25–45 years, and predominantly of German nationality or origin (67–82%). The majority were men who have sex with men (99%). With regard to HIV infections, PrEP proved to be highly effective. There were only isolated cases of HIV infections (HIV incidence rate 0.08/100 person years); in most cases the suspected reason was low adherence. The incidences of chlamydia, gonorrhea, and syphilis did not increase but remained almost the same or even decreased. A need for information on PrEP for people in trans*/non-binary communities, sex workers, migrants, and drug users emerged. Needs-based services for target groups at increased risk of HIV are necessary.

**Discussion:**

PrEP proved to be a very effective HIV prevention method. The partly feared indirect negative influences on STI rates were not confirmed in this study. Due to the temporal overlap with the containment measures during the COVID-19 pandemic, a longer observation period would be desirable for a conclusive assessment.

## Hintergrund

Für gesetzlich Krankenversicherte mit einem substantiellen HIV-Infektionsrisiko gibt es seit September 2019 mit dem Terminservice- und Versorgungsgesetz (§ 20j Fünftes Buch Sozialgesetzbuch – SGB V) einen Anspruch auf die HIV-Präexpositionsprophylaxe (PrEP; [1]). Die PrEP ist eine effektive HIV-Präventionsmethode, bei der HIV-negative Personen ein bereits seit vielen Jahren für die HIV-Therapie zugelassenes Medikament (Tenofovirdisoproxil + Emtricitabin, TDF/FTC) einnehmen, um sich vor einer HIV-Infektion zu schützen [2–8]. Der gesetzliche Anspruch auf PrEP umfasst die Beratung, die Versorgung mit dem Arzneimittel und die empfohlenen Untersuchungen [1, 9].

Das Robert Koch-Institut (RKI) hatte den Auftrag vom Bundesministerium für Gesundheit (BMG), die Einführung des neuen Leistungsanspruchs wissenschaftlich zu begleiten und zu evaluieren. Dabei sollten die Auswirkungen der Schutzmöglichkeit auf das Infektionsgeschehen sowohl von HIV als auch auf andere sexuell übertragbare Infektionen (STI) wie Chlamydien, Gonorrhö und Syphilis untersucht sowie weitere PrEP-Bedarfe und mögliche Zugangsbarrieren erhoben werden [10]. Die Evaluation erfolgte in dem vom RKI geleiteten Forschungsvorhaben „Evaluation der Einführung der HIV-Präexpositionsprophylaxe als Leistung der Gesetzlichen Krankenversicherung“ (EvE-PrEP). Die Evaluation bezog sich auf den Zeitraum 01.09.2019 bis 31.12.2020 und die Projektlaufzeit war vom 01.01.2020 bis 31.12.2021. Die Projektleitung von EvE-PrEP lag beim RKI. Das Forschungskonsortium setzte sich zusammen aus der Deutsche Arbeitsgemeinschaft ambulant tätiger Ärztinnen und Ärzte für Infektionskrankheiten und HIV-Medizin e. V. (dagnä), dem Lehrstuhl für Medizinmanagement der Universität Duisburg-Essen, der Deutschen Aidshilfe (DAH), dem Universitätsklinikum Bonn mit der BRAHMS-Studie, dem Kompetenzzentrum für Klinische Studien Bremen (KKSB) als Vertrauensstelle für Krankenkassendaten sowie mehreren gesetzlichen Krankenkassen (Techniker Krankenkasse –TK, Wissenschaftliches Institut der Allgemeinen Ortskrankenkassen – WIdO/AOK; [11, 12]).

Die Studien- und Datenquellen, die in das Projekt einflossen, stammten aus der am RKI etablierten Surveillance von HIV und Syphilis sowie verschiedenen RKI-Studien. Darüber hinaus wurden Apothekenabrechnungsdaten und Routinedaten mehrerer gesetzlicher Krankenkassen als Sekundärdatenquellen genutzt. Eine möglichst weitgehende Beteiligung von PrEP-Nutzenden erfolgte u. a. über Befragungen im Rahmen der Checkpoint‑, BRAHMS- und PrApp-Studie. Die PrEP-Verordnenden wurden über das dagnä-Netzwerk zum PrEP-Versorgungsgeschehen befragt. Darüber hinaus erfolgte eine Beteiligung von Vertretenden aus unterschiedlichen Communitys mit potenziellem PrEP-Bedarf und -Gebrauch über einen Community-Beirat [13].

Ziel dieses Artikels ist es, die gesammelten Ergebnisse über die Auswirkungen der PrEP auf das Infektionsgeschehen sowohl von HIV als auch auf andere STI erstmalig in dieser Form zusammenzustellen, weitere PrEP-Bedarfe und mögliche Zugangsbarrieren zur PrEP aufzuzeigen sowie Verbesserungsvorschläge bei der Umsetzung und Verbreitung der PrEP zu präsentieren.

## Methoden

### Apothekenabrechnungsdaten

Die Apothekenabrechnungsdaten repräsentieren eingelöste Rezepte von Personen mit gesetzlicher Krankenversicherung (GKV; ~ 88 % der deutschen Bevölkerung; [18]). Der Anbieter (Insight Health) gibt eine Abdeckung von ~ 99 % innerhalb des GKV-Marktes an. In den anonymen aggregierten Daten sind keine Behandlungsindikation und keine Charakteristika von Personen enthalten. Ausgewertet wurde die Anzahl der monatlichen und quartalsweisen Verordnungen von TDF/FTC anhand des ATC-Codes^1^ J05AR03. Es wurden 2 Szenarien mit unterschiedlichen Annahmen zum Anteil von anlassbezogenem PrEP-Gebrauch sowie der durchschnittlichen Zahl der monatlich eingenommenen PrEP-Tabletten berechnet, um die Gesamtzahl der PrEP-nutzenden Männer, die Sex mit Männern haben (MSM) in Deutschland zum Zeitpunkt Ende Juni 2020 zu schätzen [14].

### GKV-Routinedaten

Bei den GKV-Routinedaten handelte es sich um Daten von PrEP-Nutzenden der TK und dem WIdO/AOK. Damit trugen sowohl das AOK-System als auch der Bereich der Ersatzkassen zur Datenlieferung bei. Die eingeschlossenen Krankenkassen deckten über 50 % der GKV-Versicherten in Deutschland ab [15]. Bereitgestellt wurden die Daten anonymisiert über die Vertrauensstelle am KKSB. In der Routinedatenanalyse wurde bei PrEP-Nutzenden unter anderem der Zeitraum vor und nach PrEP-Start in Bezug auf die Inzidenzen der STI Chlamydien, Gonorrhö und Syphilis untersucht. Der Datenzeitraum erstreckte sich vom 01.01.2019 bis zum 31.03.2020. Von TK und WIdO in die Analysepopulation eingeschlossen werden konnten 6615 Personen, die dort anhand des ATC-Codes J05AR03 im Datenzeitraum als PrEP-Nutzende identifiziert wurden.

### BRAHMS-Studie

Die BRAHMS-Studie (Studiennummer NCT03884816) war eine Vorstudie zu einer in Deutschland geplanten Impfstoffstudie zu HIV-Subtyp B. Darin wurden in Deutschland MSM mit erhöhten Risiken für den Erwerb einer HIV-Infektion (vorausgegangene STI oder kondomloser Analverkehr mit mehr als einem männlichen Partner mit HIV oder unbekanntem Status in den letzten 24 Wochen) systematisch bezüglich des Auftretens von HIV-Infektionen und STI untersucht sowie ausführlich zu relevanten Risikofaktoren befragt [16]. In die BRAHMS-Studie wurden 1017 MSM (Datenstand 25.06.2021) über einen Zeitraum von Juni 2018 bis April 2019 eingeschlossen. Von den Studienteilnehmern berichteten 54,0 % von PrEP-Gebrauch zum Zeitpunkt des Studieneinschlusses.

### PrApp-Studie

Die PrApp-Studie war eine anonyme Online-Befragung, für die aktive und ehemalige PrEP-Anwendende sowie Nichtanwendende von PrEP über Dating-Apps, Community-Webseiten und Checkpoints rekrutiert wurden. Ziele waren die Untersuchung von Gründen für die PrEP-Anwendung, PrEP-Bezugsquellen, PrEP-Erfahrungen und von Gründen, mit PrEP aufgehört zu haben, bzw. Barrieren für die Verwendung von PrEP. An der dritten und vierten Befragungswelle nahmen jeweils 2625 Personen und 894 Personen in den Zeiträumen 24.02.2020 bis 19.05.2020 und 02.11.2020 bis 07.01.2021 teil. Insgesamt wurden die Angaben von 3519 Teilnehmenden ausgewertet.

### Checkpoint-Studie

In der Checkpoint-Studie wurden im Zeitraum Januar 2019 bis August 2021 in Beratungs- und Testeinrichtungen aus dem Verband der DAH in deutschen Großstädten standardisiert Daten erhoben und in Kooperation mit dem RKI ausgewertet: soziodemografische Merkmale, Test-Inanspruchnahme und -Motive, sexuelle Risiken, Partnerzahlen, Substanzgebrauch im Zusammenhang mit sexuellen Handlungen, STI-Diagnosen sowie Angaben zur PrEP-Einnahme. Aus den Angaben zur PrEP-Einnahme ließen sich 5 Gruppen einteilen: keine PrEP, PrEP-Wunsch, aktuell keine PrEP mehr, PrEP bei Bedarf, tägliche PrEP. Diese ließen sich in Bezug auf HIV und andere STI sowie weitere Charakteristika vergleichen. Insgesamt lagen 47.186 Fragebögen vor, hiervon 22.704 aus dem Jahr 2019, 13.650 aus 2020 sowie 10.832 aus dem Zeitraum Januar bis August 2021. Für die PrEP-bezogenen Auswertungen wurden nach Ausschluss von Frauen, heterosexuellen Männern und Visiten ohne HIV- und Syphilis-Testungen 14.553 Fragebögen ausgewertet.

### National Evaluation of PrEP Outcomes and STIs (NEPOS)

Eine vom RKI in Zusammenarbeit mit der dagnä konzipierte Studie erhob bei 47 HIV-Schwerpunktzentren im dagnä-Netzwerk deutschlandweit retrospektiv anonymisierte Daten zum PrEP-Gebrauch, zur Verbreitung, Testung und Therapie von STI und weiteren relevanten Fragestellungen (Substudie NEPOS). Zur Erhebung der Daten bei den HIV-Schwerpunktzentren wurde vom RKI eine freie Software in C++ programmiert, mit der die Daten anonymisiert, elektronisch erhoben und sicher übermittelt wurden. In NEPOS wurden Daten von 4620 PrEP-Nutzenden mit einer Gesamtbeobachtungszeit von 5132 Personenjahren (PJ; Median 451 Tage; IQR: 357–488) für den Beobachtungszeitraum 01.09.2019 bis 31.12.2020 ausgewertet [17].

Des Weiteren erfolgte eine gesonderte online-basierte retrospektive Befragung über die RKI-eigene Software Voxco bei HIV-Schwerpunktzentren im dagnä-Netzwerk für den Zeitraum 01.09.2019 bis 31.12.2020. Ziel war es, zusätzliche Aspekte im PrEP-Versorgungsalltag auf Zentrumsebene zu beleuchten und ein Meinungsbild zur Versorgungslage einzuholen. Von 43 der 47 (91 %) in der NEPOS-Substudie eingeschlossenen HIV-Schwerpunktzentren wurden die Fragen vollständig beantwortet [18, 19].

### Community-Beirat

Für die Teilnahme am Beirat wurden unterschiedliche Communitys und Verbände angefragt, von denen letztlich folgende Community-Vertretungen für die Mitarbeit im Projekt EvE-PrEP gewonnen wurden: VIA e. V./Afrikaherz/AGHNiD, Berufsverband erotische und sexuelle Dienstleistungen (BesD), DAH, HILFE-FÜR-JUNGS e. V./subway, Infoportal und PrEP-Nutzenden-Vertretung (prep.jetzt) sowie ein Mitarbeiter vom Checkpoint BLN und gleichzeitiger PrEP-User. Im Rahmen von Treffen des Community-Beirats fanden vorstrukturierte Diskussionen bestimmter Fragen zur PrEP statt. Im Anschluss an die Beiratstreffen wurden die Ergebnisse jeweils in Form eines Reports auf der Projekthomepage veröffentlicht [20–22].

## Ergebnisse

### Charakterisierung der PrEP-Nutzenden

In den *GKV-Routinedaten*, der *PrApp-Studie* und der *NEPOS-Erhebung* waren die PrEP-Nutzenden zum allergrößten Teil männlich (98–99 %), zum Großteil im Alter zwischen 25–45 Jahren (Median 35–38 Jahre) und überwiegend deutscher Staatsangehörigkeit oder Herkunft (67–82 %; [23]). Der Großteil der PrEP-Nutzenden in der *NEPOS-Erhebung* zählte zur Gruppe der MSM (99 %; [17]). Anhand der *GKV-Routinedaten* konnte beobachtet werden, dass die PrEP-Nutzenden vermehrt aus großstädtischen Gebieten stammten. Laut *Checkpoint*- und *PrApp*-Daten war die überwiegende Mehrheit der PrEP-Nutzenden ebenfalls MSM. In anteilsmäßig geringerem Umfang und in niedrigen absoluten Zahlen befanden sich aber auch trans* und genderdiverse Personen unter den PrEP-Nutzenden [23–25].

### Schätzung der Anzahl der PrEP-Nutzenden in Deutschland

Auf Grundlage der *Apothekenabrechnungsdaten* und Annahmen zum Anteil mit anlassbezogenem PrEP-Gebrauch aus der PrApp-Studie schätzten wir zwischen 15.600–21.600 PrEP-nutzende MSM in Deutschland zum Zeitpunkt Ende Juni 2020 [14]. Es zeigten sich deutliche regionale Unterschiede beim PrEP-Gebrauch mit einer gehäuften PrEP-Nutzung in Berlin (29 %), Nordrhein-Westfalen (20 %) und Bayern (14 %), gefolgt von Hessen (8 %), Baden-Württemberg (6 %) und Hamburg (6 %). Eine Korrelation der regionalen Verteilung hinsichtlich PrEP-Nutzungsabsicht, der tatsächlichen PrEP-Nutzung sowie der PrEP-Verordnenden ergab Hinweise darauf, dass der PrEP-Bedarf v. a. in Gebieten, in denen es weniger PrEP-Verordnende gibt, nicht ausreichend gedeckt war.

Größte Unsicherheit bei der Bestimmung der Zahl der PrEP-Nutzenden ist die Abschätzung der Zahl der unregelmäßigen und gelegentlich Nutzenden. Erschwerend kommt hinzu, dass die befragten PrEP-Nutzenden, HIV-Schwerpunktzentren und der EvE-PrEP-Community-Beirat angaben, dass ein relevanter Anteil der täglichen PrEP-Nutzenden während der COVID-19-Lockdowns auf anlassbezogenen PrEP-Gebrauch umgestiegen ist [12, 21].

### Art der Einnahme

In der *NEPOS-Erhebung* wurde der Modus der PrEP-Einnahme für 81 % als täglich und für 19 % als anlassbezogene PrEP angegeben [17]. Sehr ähnlich waren die Anteile in der *PrApp-Studie* und *BRAHMS-Studie*; hier gaben jeweils 81 % und 83 % der PrEP-Nutzenden eine tägliche PrEP-Einnahme an. Jeweils 19 % und 17 % verwendeten PrEP anlassbezogen.

Die Betrachtung des Quotienten aus Anzahl verordneter Tabletten zur Zeit unter PrEP bei *NEPOS* und *GKV-Routinedaten* ergab geringere Anteile mit adhärenter täglicher PrEP (~ 77 %). Es könnte sich hierbei insbesondere bei den *GKV-Routinedaten* sowohl um anlassbezogene Einnahme als auch um eine tägliche PrEP mit geringerer Adhärenz handeln [23].

In den *GKV-Routinedaten* fand sich in den 5 größten Städten Deutschlands ein statistisch signifikant höherer Anteil von nicht täglich PrEP-Nutzenden als in den anderen Orten. Diese Tendenz zeigte sich auch in den *NEPOS-Daten*, insbesondere für Berlin. Diese Beobachtung könnte dafür sprechen, dass das Spektrum der PrEP-Nutzenden in diesen Großstädten breiter ist und im Unterschied zu den übrigen Regionen auch Personen mit etwas geringeren sexuellen Risiken umfasst.

### Anteil, Gründe und Zeitpunkt von PrEP-Pausen und PrEP-Abbrüchen

In der *NEPOS-Erhebung* lagen der Anteil der berichteten PrEP-Pausen und PrEP-Abbrüche, definiert als Unterbrechung > 4 Wochen, bei 10 % und 13 %. Allerdings war die Zuordnung einer PrEP-Unterbrechung und eines PrEP-Abbruchs bei anlassbezogener Einnahme mitunter schwierig. Insofern könnten die Anteile von PrEP-Abbrüchen oder PrEP-Pausen möglicherweise höher liegen und die dokumentierten Pausen und Abbrüche vielmehr eine untere Grenze darstellen. Sensitivitätsanalysen in Bezug auf die einer Nachbeobachtung entgangenen Fälle („lost to follow-up“) zeigten PrEP-Abbruchraten um die 23 %. Insbesondere im Jahr 2020, in dem es durch die COVID-19-Pandemie zu zahlreichen (sozialen) Einschränkungen kam, erscheinen Anteile von rund einem Viertel PrEP-Abbrüche bzw. einem Drittel anlassbezogene PrEP-Nutzungen plausibler [17]. Des Weiteren entfielen 49 % der PrEP-Pausen auf die Monate März und April 2020 und damit in die Zeit des ersten Lockdowns. Den Zusammenhang mit der COVID-19-Pandemie zeigten auch die Angaben zu den Gründen für PrEP-Pausen und PrEP-Abbrüche: In der *NEPOS-Erhebung* waren die häufigsten Gründe die COVID-19-Pandemie (38 %), gefolgt von Patient*innenwunsch (17 %) sowie Änderung des Sexualverhaltens (12 %). Nebenwirkungen spielten eine untergeordnete Rolle und wurden in nur 3 % der Fälle angegeben ebenso wie Begleiterkrankungen (4 %), medizinische Interaktionen (0,2 %) und die Zunahme von STI (1 %; [17, 23]).

### Anzahl der HIV-Neuinfektionen in Zusammenhang mit PrEP

In der *NEPOS-Erhebung* lag der Anteil der HIV-Infektionen im Zusammenhang mit PrEP bei 0,087 % (4/4620), die Inzidenzrate betrug 0,078/100 PJ. Bei 2 der 4 Fälle wurden als Gründe für die HIV-Infektion Probleme mit Adhärenz und unregelmäßige Einnahme berichtet. Bei einem Fall fand die HIV-Infektion wahrscheinlich vor PrEP-Start statt. Bei einem weiteren Fall wurde eine Resistenz gegen FTC nachgewiesen und von der betroffenen Person die tägliche adhärente Einnahme angegeben. Der Quotient aus verordneten Tabletten zur PrEP-Zeit deutete jedoch auch bei diesem Fall auf eine geringere Adhärenz hin [17].

In der *Checkpoint-Studie* lag der Anteil der reaktiven HIV-Befunde insgesamt bei 0,66 % (96/14.553). Bei Personen ohne PrEP und ohne PrEP-Wunsch war dieser Anteil mit 0,70 % etwas höher (74/10.550), bei Personen mit PrEP-Wunsch mit 1,18 % (17/1440) noch einmal höher und bei Personen, die aktuell keine PrEP mehr verwendeten, mit 1,31 % (2/153) am höchsten. Allerdings ist Letztere die kleinste Gruppe und die Aussagen sind daher nicht generalisierbar. Bei Personen, die PrEP bei Bedarf einnahmen, lag der Anteil der HIV-reaktiven Befunde bei 0,31 % (2/654) und sank nochmals auf 0,06 % bei täglicher Einnahme (1/1756). Die konsequente Einnahme einer PrEP war also mit einem deutlich verminderten Risiko einer HIV-Infektion verbunden (Tab. 1). Insgesamt hatten in der *Checkpoint-Studie* 7 Personen, die seit einem vorangegangenen negativen HIV-Test von einer PrEP-Einnahme berichteten, ein reaktives HIV-Testergebnis erhalten. Wie in der *NEPOS-Erhebung* zeigte sich auch hier, dass ein Teil der Infektionen (2/6 mit Angabe hierzu) wahrscheinlich auf nicht korrekte PrEP-Einnahme zurückzuführen war. Weiterhin gaben auch hier 4 von 7 Personen PrEP-Einnahme bei Bedarf an. In mindestens einem Fall mit einmonatiger PrEP-Dauer wäre das Vorliegen einer unentdeckten HIV-Infektion zu Beginn der PrEP-Einnahme möglich. Einnahmefehler oder Infektionen mit resistenten Varianten kämen als weitere mögliche Ursachen für die Infektionen infrage.

**Table 1:** 

	HIV	Syphilis reaktiv	Syphilis positiv	Gonokokken (NG)	Chlamydien (CT)	Co-Infektion NG/CT
Anzahl	96	1482	204	828	975	151
Keine PrEP	0,70 %	6,9 %	1,0 %	5,3 %	6,9 %	0,8 %
PrEP-Wunsch	1,18 %	10,8 %	2,2 %	9,4 %	9,3 %	2,1 %
Aktuell nicht	1,31 %	16,3 %	0,7 %	9,0 %	10,5 %	–
Bei Bedarf	0,31 %	20,5 %	2,5 %	10,3 %	10,8 %	2,3 %
Täglich	0,06 %	24,9 %	2,8 %	10,9 %	11,3 %	2,7 %
Gesamt	0,66 %	10,2 %	1,4 %	6,8 %	8,0 %	1,2 %

*STI* sexuell übertragbare Infektionen, *PrEP* HIV-Präexpositionsprophylaxe, *NG* Neisseria gonorrhoeae, *CT* Chlamydia trachomatis

### STI unter PrEP sowie Krankheitslast von STI mit und ohne PrEP-Gebrauch

#### Häufigkeit von Chlamydien, Gonokokken und Syphilis bei PrEP-Nutzenden

**Figure 1:**
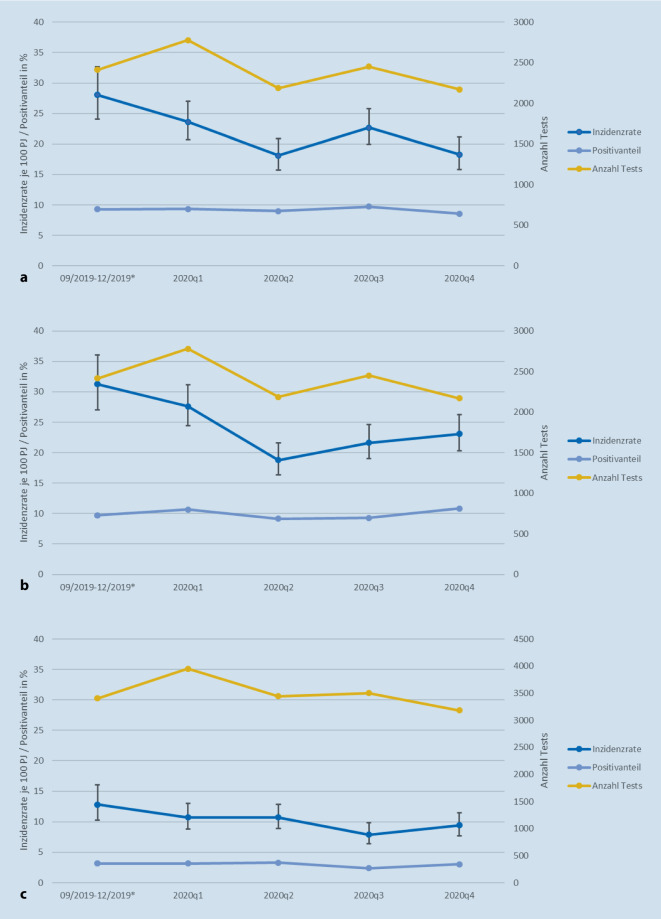

In der *NEPOS-Erhebung* lag die STI-Inzidenzrate unter HIV-PrEP im gesamten Untersuchungszeitraum (01.09.2019–31.12.2020) für Chlamydien bei 21,6/100 PJ (95 %-Konfidenzintervall (KI) 20,2–22,9), für Gonorrhö bei 23,7/100 PJ (95 % KI 22,4–25,2) und für Syphilis bei 10,1/100 PJ (95 % KI 9,2–11,0). Die Gesamtinzidenz für irgendeine der untersuchten STI (Chlamydien, Gonorrhö oder Syphilis) betrug 55,4/100 PJ (95 % KI 53,2–57,5). In der Erhebung war sowohl für die STI-Gesamtinzidenz als auch für die Inzidenz der einzelnen STI keine Zunahme, sondern eine signifikante Abnahme zu verzeichnen. Insgesamt ergab sich über den Untersuchungszeitraum eine Abnahme der Inzidenzen von Chlamydien um 34,8 %, von Gonorrhö um 26,1 % und von Syphilis um 26,5 %. Die Gesamtinzidenz für irgendeine der untersuchten STI ging während des Untersuchungszeitraums um 29,6 % zurück (Abb. 1).

Im Verlauf zeigten sich Unterschiede in der Anzahl der Tests, mit der geringsten Anzahl für Chlamydien/Gonorrhö im 2. und 4. Quartal 2020 (*n* = 2186 und *n* = 2171) und für Syphilis im 4. Quartal (*n* = 3183). Die höchste Testanzahl war im 1. Quartal 2020 zu verzeichnen (Chlamydien/Gonorrhö *n* = 2779; Syphilis *n* = 3952). Insgesamt war die Anzahl der Syphilistests (*n* = 18.598) deutlich höher als die Anzahl der Chlamydien‑/Gonorrhötests (*n* = 12.789).

Der Positivanteil war über den Beobachtungszeitraum relativ konstant und schwankte um einen Mittelwert von 9,2 %, 9,9 % und 3,0 % für Chlamydien, Gonorrhö und Syphilis (Abb. 1).

Im Rahmen der *PrApp-Studie* berichteten 42,1 % der Personen, jemals eine Gonorrhö-Diagnose erhalten zu haben, 39,9 % von einer Chlamydien-Diagnose und 27,4 % von einer Syphilis-Diagnose. Wiederholte Diagnosen dieser STI innerhalb der letzten 12 Monate traten nur bei einer Minderheit der PrEP-Nutzenden auf.

#### Häufigkeit von STI mit und ohne PrEP-Gebrauch

Die Einschlusskriterien der *BRAHMS-Studie* entsprachen weitgehend den Kriterien zur Verschreibung einer PrEP (vorausgegangene STI oder kondomloser Analverkehr). Zu Beginn der Studie wurde bei einem Anteil von 35,5 % der Teilnehmenden mindestens eine Infektion mit Chlamydien, Gonokokken, Mykoplasmen, Treponema pallidum (Syphilis-Erreger), Hepatitis-C-Virus oder HIV diagnostiziert, am häufigsten mit Mykoplasmen (19,0 %), gefolgt von Chlamydien (12,8 %), Gonokokken (10,1 %) und Treponema pallidum (3,5 %). In 85 % der Fälle waren diese asymptomatisch und wurden nur durch das Screening entdeckt [16].

In Bezug auf die STI-Prävalenz beim Screening für die Aufnahme in die Kohorte von MSM mit erhöhtem HIV-Infektionsrisiko ergab sich in einem adjustierten Modell kein signifikanter Unterschied für Mykoplasmen, Chlamydien und Gonorrhö bei PrEP-Nutzenden und Personen, die keine PrEP nahmen.

In der *Checkpoint-Studie* wurden bei PrEP-Nutzenden höhere Prävalenzen behandlungsbedürftiger STI gefunden als bei Personen ohne PrEP. Während bakterielle STI bei Personen mit täglicher PrEP am häufigsten waren, waren HIV-Infektionen bei Personen mit täglicher PrEP-Einnahme erwartungsgemäß am niedrigsten (Tab. 1).

Die Ergebnisse in Bezug auf PrEP-Nutzung und STI deuten auf ein unterschiedliches Risiko von Personen ohne und mit PrEP-Nutzung hin, dazu gehören bspw. mehr Partner, mehr kondomloser Sex sowie Kontakte innerhalb von Netzwerken mit höheren STI-Raten.

In der *Checkpoint-Studie* ergaben multivariable Analysen der Risikofaktoren für STI (Chlamydien, Gonokokken, Syphilis), dass bei allen 3 Infektionen neben der Anzahl der Sexualpartner*innen auch ein sexualisierter Substanzgebrauch (Chemsex) eine Rolle spielte. Bei Chlamydien und Syphilis war ein zusätzlicher, von den Partnerzahlen unabhängiger Zusammenhang mit dem PrEP-Status, insbesondere bei täglicher PrEP, zu beobachten. Das Alter zeigte bei Chlamydien und Gonokokken eine mit steigendem Alter sinkende Prävalenz (2–3 % weniger pro zusätzliches Lebensjahr). Dies könnte mit erworbener Immunität zusammenhängen.

**Figure 2:**
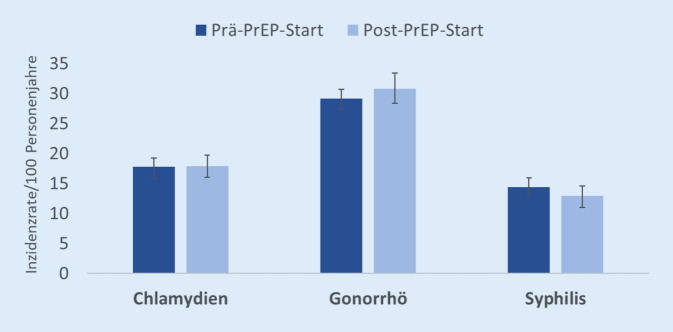

In der Analyse von *GKV-Routinedaten* wurde bei PrEP-Nutzenden der Zeitraum vor und nach PrEP-Start in Bezug auf die Inzidenzen der STI Chlamydien, Gonorrhö und Syphilis untersucht. Der Beobachtungszeitraum in der Routinedatenanalyse endete am 31.03.2020, wodurch ein nur geringer Einfluss der COVID-19-Pandemie angenommen werden kann. Die durchschnittliche Inzidenz für Chlamydien-Infektionen vor und ab der ersten PrEP-Abgabe veränderte sich nur im Nachkommabereich. Auch bei den Infektionen mit Gonokokken kam es lediglich zu einer sehr geringen Zunahme. Bei der Syphilis zeigte sich eine geringe Abnahme der Mittelwerte von Prä-PrEP-Start auf Post-PrEP-Start (Abb. 2).

Keine der Veränderungen in den berechneten STI-Raten im Vergleich der Zeiträume von vor und ab der ersten PrEP-Abgabe war statistisch signifikant (Wilcoxon-Test für verbundene Stichproben, statistisch signifikant *p* < 0,05). Längere Beobachtungsverläufe in Routinedaten wären hilfreich, um die Auswirkungen der HIV-PrEP auf Testung und Diagnose von STI noch valider zu bewerten.

### Gründe für Nichtinanspruchnahme der PrEP

In der *NEPOS-Erhebung* waren die häufigsten Gründe für die Nichteinleitung der PrEP aus Sicht der HIV-Schwerpunktzentren (*n* = 43): individueller Patient*innenwunsch (72 %), Angst vor Nebenwirkungen (56 %), fehlende Indikation (51 %), feste Partnerschaft (40 %) und die COVID-19-Pandemie (26 %). Interessant ist der hohe Anteil, den die Angst vor Nebenwirkungen einnahm, da in der *NEPOS-Erhebung* nur zu einem sehr geringen Anteil (~ 3 %) Nebenwirkungen als Grund für PrEP-Unterbrechungen und -Abbruch genannt wurden [18, 19].

In der *PrApp-Studie* wurden unter den Nicht-PrEP-Nutzenden, die aufgrund weiterer Angaben eine PrEP-Indikation hatten, folgende Gründe am häufigsten genannt: Angst vor Nebenwirkungen (53 %), PrEP ist zu aufwendig (35 %), möchte nicht mit Ärzt*in über Sex reden (31 %), eigenes HIV-Risiko wird nicht hoch genug eingeschätzt (25 %) sowie kann keine Ärzt*in finden, die PrEP verschreibt (22 %). Die Angst vor Nebenwirkungen war hier der häufigste Grund. Eine weitere Barriere schien die Verfügbarkeit von HIV-PrEP verordnenden Ärzt*innen darzustellen.

### PrEP für weitere Personengruppen, Zugangsbarrieren und Verbesserungspotenzial bei der GKV-PrEP

Wie unsere Auswertungen nahelegen, ist die PrEP in Deutschland bisher hauptsächlich bei MSM und nur in geringem Maße in weiteren Gruppen verbreitet. Im engen Austausch und Treffen mit verschiedenen Communitys, die in EvE-PrEP vertreten waren, wurde ebenfalls berichtet, dass es kaum PrEP-Gebrauch außerhalb der MSM-Community gibt. Als mögliche Gründe hierfür wurden verschiedene Zugangsbarrieren diskutiert, die eine unterschiedlich starke Rolle in den Communitys spielen, wie das Fehlen einer Krankenversicherung, Versorgungsprobleme in ländlichen Regionen durch das Fehlen von HIV-Schwerpunktzentren, der Zugang von Frauen zur PrEP sowie für Personen in der Sexarbeit der vermeintliche Widerspruch zur sogenannten Kondompflicht laut aktuellem Prostituiertenschutzgesetz.

„Größte Zugangsbarriere ist nicht vorhandene Krankenversicherung“ (VIA e. V./Afrikaherz, BeSD e. V., HILFE-FÜR-JUNGS e. V./subway; sinngemäß).

„Auch mit EU-Versicherungskarte normalerweise keine Kostenübernahme für PrEP“ (Checkpoint BLN).

„Zunächst muss Versicherung in der GKV bestehen. Dringender Wunsch nach besserem Zugang zur GKV für Sexarbeiter*innen!“ (BeSD e. V; [20–22]).

Die COVID-19-Pandemie hat den Fokus von der PrEP weggelenkt, einschließlich der Bekanntmachung der PrEP in weiteren Gruppen über MSM hinaus. Prekäre Lebenslagen sowie die genannten Zugangsbarrieren haben sich zum Teil verstärkt.

„Arbeiten unter dem Tätigkeitsverbot für Sexarbeitende ohne Einkommens-Alternative mit starker Gefährdung für die Gesundheit (auch psychische z. B. durch gesellschaftliche Ächtung usw.) verbunden“ (BeSD e. V.).

„Behörden im Lockdown für Monate geschlossen, keine Aufenthaltstitel oder Verlängerungen oder verzögerte Bearbeitung von Asylbegehren. Die Gesamtsituation ist noch schlechter geworden durch unsicheren Aufenthaltsstatus und damit fehlende Krankenversicherung. Soziale Isolation, Einschränkung der Bewegungsfreiheit gefährdeten die psychische Gesundheit“ (VIA e. V./Afrikaherz; [20–22]).

In den Treffen wurde aber deutlich, dass es in den weiteren Communitys über MSM hinaus durchaus PrEP-Bedarf gibt, zunächst jedoch ein hoher Aufklärungs- und Informationsbedarf bestehe, bspw. zu den Themen Nebenwirkungen, Langzeitfolgen und Entstigmatisierung. Dies betrifft zum Beispiel die afrikanische Community, bei der grundsätzlich ein gewisser PrEP-Bedarf von den Community-Vertretenden gesehen wurde.

„Afrikanische Community ist eine kleine, aber von HIV stark betroffene Community. Vor der PrEP-Einnahme würde zunächst die Aufklärung stehen. Diese könnte stattfinden in Beratungs- und Gesundheitsstellen der afrikanischen Community. Kulturelle Aspekte müssten adressiert werden“ (VIA e. V./Afrikaherz; [20–22]).

Des Weiteren wurde deutlich, dass bei Personen aus trans* und nichtbinären Communitys sowie bei Sexarbeitenden ein potenzieller PrEP-Bedarf besteht, zumindest ein hoher Bedarf nach mehr Informationen über PrEP und den Zugang zur PrEP.

Die *Checkpoint-Daten* zeigten bei trans* und genderdiversen Personen sowohl HIV-Infektionsrisiken und HIV-Inzidenzen in ähnlicher Größenordnung wie bei MSM als auch den Wunsch und die Bereitschaft, PrEP einzunehmen (Tab. 2 im Anhang). Bedarfsgerechte Angebote und Informationen sollten daher für diese Personengruppen bereitgestellt werden.

Verbesserungspotenzial sieht der Community-Beirat weiterhin bei den zur Verordnung der GKV-PrEP nötigen fachlichen Voraussetzungen, wie der Weiterbildung für Ärzt*innen. Diese sollten niedrigschwelliger gestaltet sein und beispielsweise um Online-Angebote ergänzt werden, damit auch in ländlichen Regionen eine PrEP-Versorgung gewährleistet werden kann.

## Diskussion

Die PrEP-Nutzenden waren zum größten Teil männlich (98–99 %), zum Großteil im Alter zwischen 25–45 Jahren (Median 35–38 Jahre) und überwiegend deutscher Staatsangehörigkeit oder Herkunft (67–82 %; [24]). Der Großteil der PrEP-Nutzenden in NEPOS zählte zur Gruppe der MSM (99 %; [17]). Zum Stand Ende Juni 2020 schätzten wir zwischen 15.600–21.600 PrEP-nutzende MSM in Deutschland [14]. Nach neueren Schätzungen aus der Surveillance der Versorgung mit der HIV-PrEP in Deutschland (Projekt PrEP-Surv) gibt es mit Stand Ende 2022 rund 32.000 PrEP-Nutzende in Deutschland [26]. Es zeigten sich deutliche regionale Unterschiede beim PrEP-Gebrauch mit einer gehäuften PrEP-Nutzung in großstädtischen Gebieten, angeführt von Berlin.

In Bezug auf HIV-Infektionen erwies sich die PrEP im klinischen Alltag als hocheffektiv. Es fanden nur vereinzelt HIV-Infektionen in Zusammenhang mit PrEP-Nutzung statt, in den meisten Fällen war der vermutete Grund eine geringe Adhärenz. Ansätze zur Unterstützung und Sicherstellung der Adhärenz wären daher wichtig. Die beobachteten HIV-Inzidenzen lagen im Bereich vergleichbarer Studien zur Wirksamkeit der HIV-PrEP oder sogar darunter [2–4, 27–30]. Ob die PrEP genügend und die richtigen Personen erreicht, um die HIV-Inzidenz mittel- und längerfristig nachhaltig zu reduzieren, lässt sich aufgrund des Einflusses der COVID-19-Pandemie noch nicht beurteilen. Die Zahl der HIV-Neudiagnosen sowie die geschätzte Zahl der HIV-Neuinfektionen nahm in Deutschland und in der Gruppe der MSM in den letzten Jahren kontinuierlich ab [31]. Der Rückgang der HIV-Neudiagnosen in Berlin, wo ein großer Teil der PrEP-Verordnungen erfolgte, könnte allerdings ein Indiz für einen epidemiologischen PrEP-Effekt auf die sinkenden HIV-Neuinfektionszahlen darstellen [31].

Eine Sorge im Zusammenhang mit der PrEP-Nutzung ist eine Zunahme von STI. In einigen Studien wurde ein Anstieg von STI mit vermehrter PrEP-Nutzung in Verbindung gebracht [32–34]. Zu beachten ist, dass die Analyse des Zusammenhangs zwischen PrEP und STI wesentlich davon beeinflusst wird, dass PrEP-Nutzende mehr Sexpartner im Allgemeinen und mehr Sexpartner mit kondomlosem Analverkehr haben als Personen, die keine PrEP nutzen. Außerdem werden PrEP-Nutzende deutlich häufiger auf STI getestet als Personen, die keine PrEP nutzen. In unserer Untersuchung nahm die STI-Inzidenz (Chlamydien, Gonorrhö, Syphilis) über den Studienverlauf nicht zu, sondern blieb nahezu gleich (GKV-Routinedaten) oder ging sogar zurück (NEPOS). Die Stärke der GKV-Routinedatenanalyse war die Beobachtung derselben Personen vor und nach PrEP-Start, bei der sich keine signifikante Zunahme der STI-Inzidenzen zeigte. Darüber hinaus wird der Einfluss der COVID-19-Pandemie in den GKV-Routinedaten als vernachlässigbar eingeschätzt, da die Beobachtung im März 2020 endete. Die Ergebnisse für NEPOS lassen sich hingegen nicht klar vom Einfluss der COVID-19-Pandemie trennen. Allerdings zeigte sich bereits im ersten Quartal 2020 eine Abnahme der STI-Inzidenzen. Dennoch müssen bei der Bewertung des Verlaufs der HIV- und STI-Inzidenzen im Jahr 2020 eine Reihe von Faktoren berücksichtigt werden, zu denen eine Änderung des Sexualverhaltens sowie eine geringere Verfügbarkeit von Test- und Präventionsangeboten und eine geringere Inanspruchnahme der Gesundheitsversorgung gehören. Längere Analysen im weiteren Verlauf sind notwendig, um die Auswirkungen der HIV-PrEP auf Testung und Diagnose von STI und HIV mit mehr Validität zu bestimmen und zu bewerten.

Befragungen bei den HIV-Schwerpunktzentren zeigten einen deutlichen Einfluss der COVID-19-Pandemie auf PrEP-Einnahme, -Unterbrechungen und -Nachfrage. So gaben 76 % der HIV-Schwerpunktzentren einen Rückgang der PrEP-Nachfrage im Zuge des ersten Lockdowns an [12]. Auch die Gründe für einen PrEP-Abbruch oder PrEP-Pausen hingen zumeist mit der COVID-19-Pandemie zusammen. Rund 50 % der Unterbrechungen entfielen auf den ersten pandemiebedingten Lockdown im März und April 2020 [17].

Ein häufiger Grund, weshalb die PrEP nicht eingeleitet wurde, war die Angst vor Nebenwirkungen. Bei den Gründen für Unterbrechung oder Abbruch der PrEP wurden Nebenwirkungen hingegen selten angegeben, womit die Angst vor Nebenwirkungen deutlich stärker ausgeprägt war als deren dokumentierte Häufigkeit [17–19]. Hieraus ergeben sich sowohl ein Aufklärungsbedarf, um an PrEP interessierten Menschen eine informierte, faktenbasierte Entscheidung zu ermöglichen, als auch ein Potenzial für eine stärkere PrEP-Nutzung bei Menschen mit erhöhtem HIV-Risiko.

Im Austausch mit dem Community-Beirat zeigte sich ein Informationsbedarf zur PrEP für Personen in verschiedenen Communitys. Notwendig wären bedarfsgerechte Angebote und Informationen zur PrEP für Zielgruppen mit erhöhtem HIV-Risiko, wie es sie in anderen Ländern (USA, Australien, Frankreich) gibt, z. B. für Personen innerhalb der trans* und nichtbinären Communitys, für Sexarbeitende und für Personen aus der afrikanischen Community [20–22]. Personen, die intravenös Drogen konsumieren, gehören ebenfalls prinzipiell zu den PrEP-Anspruchsberechtigten in Deutschland; es gibt aber bisher kaum PrEP-Nutzende in dieser Gruppe. Eine Publikation aus Schottland zeigt, dass Personen, die intravenös Drogen konsumieren, durch gezielte persönliche Ansprache sehr wohl zum PrEP-Gebrauch motivierbar sind und auch eine gute Adhärenz erreichen [35]. Allerdings waren die PrEP-Beratung und Begleitung der Drogen gebrauchenden Menschen sehr personalintensiv und könnten in Deutschland wahrscheinlich nur über niedrigschwellige Drogenhilfeeinrichtungen erfolgen, die dafür entsprechend qualifiziertes Personal benötigten.

Eine standardisierte Erhebung von HIV-Infektionsrisiken und eine aktive Beratung und Information über Präventionsmöglichkeiten, inklusive der PrEP-Einnahme, sollten fester Bestandteil in der ärztlichen Beratung bei Personen aus Gruppen mit erhöhtem HIV-Infektionsrisiko werden.

Eine kontinuierliche PrEP-Beratung und Aufklärung sollten bei bereits PrEP-Nutzenden ebenfalls weiterhin gewährleistet und stetig wiederholt werden.

Damit die genannten wesentlichen Aufgaben der sexuellen Bildung geleistet werden können, müssen Gelder bereitgestellt werden und die Finanzierung gesichert sein. Außerdem gab es Hinweise, dass der PrEP-Bedarf in ländlichen Regionen, in denen es weniger PrEP-Verordnende gibt, nicht ausreichend gedeckt war und dass auch in den Großstädten viele MSM von sich aus keinen PrEP-Bedarf äußerten, obwohl eine PrEP-Indikation vorlag [14]. Angesichts der hohen Konzentration des PrEP-Versorgungsgeschehens auf die 5 größten Städte in Deutschland sowie der identifizierten Barrieren, trotz Indikation keine PrEP zu nutzen (für ca. 35 % zu hoher Aufwand, PrEP zu erhalten, bei ca. 22 % keine Verordnenden verfügbar), muss davon ausgegangen werden, dass eine bedarfsgerechte Versorgung noch nicht flächendeckend erreicht ist. Um die PrEP-Versorgung zu sichern und auszuweiten, sind Konzepte und Anreize wichtig. Dazu gehört sicherlich auch, die Voraussetzungen zur Verordnung der GKV-PrEP niedrigschwelliger zu gestalten.

## Fazit

Insgesamt ist die PrEP eine sehr wirkungsvolle HIV-Präventionsmethode. HIV-Infektionen in Zusammenhang mit PrEP traten nur vereinzelt auf und die befürchtete Zunahme von STI hat sich in dieser Untersuchung bisher nicht bestätigt. Allerdings braucht es zur umfassenderen Beurteilung längere Beobachtungszeiträume. Das RKI wird daher im Anschluss an die PrEP-Evaluation ab dem Jahr 2022 im vom BMG finanzierten Projekt „Surveillance der Versorgung mit der HIV-Präexpositionsprophylaxe innerhalb der GKV in Deutschland“ (PrEP-Surv) die Verstetigung eines Monitorings der Versorgung mit der HIV-PrEP in Deutschland etablieren [36].

Bisher wird die PrEP fast ausschließlich von Personen aus der Gruppe der MSM verwendet. Es zeigten sich aber darüber hinaus PrEP-Bedarfe in weiteren Gruppen. Um das Potenzial der PrEP als Präventionsmethode erschließen zu können, bleibt es wichtig, allen Personen mit Bedarf die PrEP zugänglich zu machen.

## Anhang

**Table 2:**

Geschlecht/sexuelle Orientierung	Nicht HIV-reaktiv	HIV-reaktiv	Gesamt
Männer, die Sex mit Männern haben	14.908	122 (0,81 %)	15.030
Heterosexuelle Männer	4841	5 (0,10 %)	4846
Frauen	5563	5 (0,09 %)	5568
Nichtbinäre Menschen	864	7 (0,80 %)	871
Trans-Männer	169	1 (0,59 %)	170
Trans-Frauen	193	2 (1,03 %)	195
Gesamt	26.538	142 (0,53 %)	26.680

## Footnotes

1 ATC steht für die **a**natomisch-**t**herapeutisch-**c**hemische Einordnung von Wirkstoffen und Arzneimitteln und ist eine international anerkannte Klassifikation und Zuordnung von Medikamenten und ihren Wirkstoffen in zusammengehörige Wirkstoffgruppen. (siehe https://www.gelbe-liste.de/atc).
